# The Effects of Freeze-Thaw and UVC Radiation on Microbial Survivability in a Selected Mars-like Environment

**DOI:** 10.3390/microorganisms10030576

**Published:** 2022-03-07

**Authors:** Daniel Keaney, Brigid Lucey, Noreen Quinn, Karen Finn

**Affiliations:** 1 Department of Biological Sciences, Munster Technological University, Bishopstown, T12 P928 Cork, Ireland; daniel.keaney@mycit.ie (D.K.); brigid.lucey@mtu.ie (B.L.); 2 Department of Mathematics, Munster Technological University, Bishopstown, T12 P928 Cork, Ireland; noreen.quinn@mtu.ie; 3 Department of Analytical, Biopharmaceutical and Medical Sciences, Galway-Mayo Institute of Technology, Old Dublin Road, H91 T8NW Galway, Ireland

**Keywords:** astrobiology, bacterial resistance, temperature cycling, UV resistance, *Paraburkholderia fungorum*, *Deinococcus radiodurans*

## Abstract

The purpose of this study was to determine survivability of *Escherichia coli*, *Deinococcus radiodurans* and *Paraburkholderia fungorum* under Mars-simulated conditions for freeze-thawing (−80 °C to +30 °C) and UV exposure alone and in combination. *E. coli* ATCC 25922, *D. radiodurans* and *P. fungorum* remained viable following 20 successive freeze-thaw cycles, exhibiting viabilities of 2.3%, 96% and 72.6%, respectively. *E. coli* ATCC 9079 was non-recoverable by cycle 9. When exposed to UV irradiation, cells withstood doses of 870 J/m^2^ (*E. coli* ATCC 25922), 200 J/m^2^ (*E. coli* ATCC 9079), 50,760 J/m^2^ (*D. radiodurans*) and 44,415 J/m^2^ (*P. fungorum*). Data suggests *P. fungorum* is highly UV-resistant. Combined freeze-thawing with UV irradiation showed freezing increased UV resistance in *E. coli* ATCC 25922, *E. coli* DSM 9079 and *D. radiodurans* by 6-fold, 30-fold and 1.2-fold, respectively. Conversely, freezing caused *P. fungorum* to exhibit a 1.75-fold increase in UV susceptibility. Strain-dependent experimentation demonstrated that freezing increases UV resistance and prolongs survival. These findings suggest that exposure to short wavelength UV rays (254 nm) and temperature cycles resembling the daily fluctuating conditions on Mars do not significantly affect survival of *D. radiodurans*, *P. fungorum* and *E. coli* ATCC 25922 following 20 days of exposure.

## 1. Introduction

Mars is a dry and barren planet with promising potential for future human colonization. Over the course of one Martian day (24.65 h), surface temperatures on Mars can vary depending on the location and the reporting source [1,2]. At the equator, temperatures at noon can reach a high of 30 °C and at the poles can reach a low of −140 °C, with an average temperature of −63 °C [3]. Present-day Mars has UV wavelengths ranging from 200–400 nm, which is comparable to the surface wavelengths that reach Earth (290–400 nm) [4]. Dissimilarly, the UV radiation environment on Mars features shorter wavelengths within the UVC (200–280 nm) and UVB (280–315 nm) spectra, with UVC being particularly biologically damaging due to its DNA disrupting capabilities [5]. This occurs as UVC energy is absorbed by DNA causing the dimerization of nucleic acid bases including cyclobutane pyrimidine species and pyrimidine (6–4) pyrimidone compounds, together with their Dewar isomers [6].

Much of the Martian surface is covered in a deep layer of dust with a primary composition of iron(III) oxide [7]. Martian soil (the fine regolith and dust comprising the surface landscape) is toxic due to the presence of high concentrations of perchlorate compounds (0.5–1%) [8,9]. Perchlorate is usually found as the anion component of a salt most often associated with cations such as ammonium, sodium, or potassium and is regarded as both a strong oxidizing anion and a toxic hazard to human health [10]. The future use and applications of bioremedial bacteria may facilitate the detoxification of Martian soils, crop cultivation and successful future colonization.

The colonization of Mars has been traditionally mapped out via a terraforming approach. The terraforming of Mars is a multi-faceted operation, which, according to Haynes and McKay, involves three main steps; the human/robotic exploration of Mars (which has been conducted through various Mars landers), the planetary engineering step; designed to warm the planet, liberate liquid water and produce a thick carbon dioxide atmosphere; and the introduction of pioneering microbial communities able to proliferate in the Martian environment [11]. While there are numerous, and technologically-feasible, answers as to how humankind would conduct the planetary engineering steps, the survivability of bioremedial microbes within a Mars-like environment is the focus of this study.

In order for bioremedial microorganisms to be successfully applied within these environments, it is imperative to understand how certain candidate microbes behave under the insult of Mars-like conditions. While the generation of terrestrial atmospheric conditions can be easily established on Mars, the immediate, and most threatening environmental insults to microbial life are daily temperature fluctuation (on average −63 °C to +30 °C) and UV radiation (UVC (200–280 nm) and UVB (280–315 nm)). Temperature cycling aims to recreate and determine the effects of daily Martian temperature fluctuations on microbial life. Similarly, in order to analyze the effects of shorter wavelengths (in particular 254 nm, given its DNA damaging capabilities) on the viability of the candidate microbes, dose-response experimentation was carried out to determine the limits of survival of the candidate strains in response to Mars-like UVC exposure.

The candidate microbes for this study were selected as follows: *Escherichia coli* ATCC 25922 and *Escherichia coli* DSM 9079 are both well-characterized microbes and functioned as the biological controls for both the freeze-thaw and UVC testing within the study. *E. coli* ATCC 25922 contains a functioning *RecA* gene, while *E. coli* DSM 9079 is a *ΔrecA* mutant that exhibits significant sensitivity to DNA damaging agents [12]. *RecA* encodes a multifunctional enzyme that plays an important role in DNA repair, homologous recombination and the induction of the SOS biomolecular response systems within the cell [13,14]. As such, this allows for the effects of environmental parameters on non-extremophilic microbes to be examined to determine the upper and lower limits of survival in phenotypically strengthened and weakened bacterial strains. Indeed, *E. coli* ATCC 25922 was used in a similar set of experiments, as reported by Gao and Leung [15], using the same 254 nm UV wavelength and the same experimental premise of freeze-thaw, both independently and in combination, thereby allowing comparison with the earlier study. However, in contrast, the current study expands on the findings of Gao and Leung and included harsher test conditions; higher doses of UVC radiation and a greater number of freeze-thaw cycles. *Deinococcus radiodurans* ATCC 13939 is a well-documented bacterium in the field of astrobiology, largely due to its radio-resistance phenotype. This phenotype is due to its multiple genome, with each cell having between four to ten genomic copies [16,17]. It has been suggested that *D. radiodurans* uses these redundant ‘multi-genomic’ copies to aid in DNA repair by means of inter-chromosomal recombination [18]. *D. radiodurans* is also a well-documented bioremedial strain, capable of remediating heavy metals such as cadmium and lead from radioactive mixed waste environments. It is also amenable to genetic recombination to further expand its bioremediation capabilities [19,20,21]. Due to both its high UV resistance and bioremedial capabilities, *D. radiodurans* was selected as an ideal control strain for UVC irradiation testing to help assess the upper limits of microbial survival when exposed to UVC energy.

*Paraburkholderia fungorum* DSM strain 17061 was chosen as little is understood about this strain within the confines of astrobiological-based bioremediation. Many space-orientated experiments focus on pathogenic strains and how space parameters influence their levels of pathogenicity, whereas the behavior of beneficial bioremedial strains under the influence of space parameters still remains to be elucidated. *P. fungorum* is capable of remediating many organics such as dibenzothiophene, fluorene, naphthalene and phenanthrene, along with polycyclic aromatic hydrocarbons other than condensed thiophenes [22].

As a result, the control and bioremedial candidate microbes were chosen for their potential suitability on Mars as useful bacteria.

This study details the responses of the candidate strains, over time, when exposed to both independent successive freeze-thaw cycles ranging from −80 °C to +30 °C, and independent UV irradiation (0–870 J/m^2^, 0–200 J/m^2^, 0–44,415 J/m^2^ and 0–50,760 J/m^2^, for *E. coli* ATCC 25922, *E. coli* DSM 9079, *P. fungorum* DSM 17061 and *D. radiodurans* ATCC 1393, respectively) followed by the combination of both freeze-thaw and UV irradiation testing. In order to account for strain-specific differences in UV tolerances, different UVC doses were used for each strain.

## 2. Materials and Methods

### 2.1. Test Micro-Organisms and Culture Preparation

*Escherichia coli* ATCC strain 25922 (American Type Culture Collection, Manassas, VA, USA), *Deinococcus radiodurans* ATCC strain 13939, *Paraburkholderia fungorum* DSM strain 17061 (DSMZ-German Collection of Micro-organisms and Cell Cultures GmbH, Braunschweig, Germany) and *Escherichia coli* DSM strain 9079 were used in this study. Cell cultures were prepared in triplicate from −80 °C 40% glycerol stock solutions. Individual colonies were picked and separately inoculated in 20 mL of nutrient broth (Formedium; NBO02). *E. coli* strains were incubated at 37 °C for 18 h, and *P. fungorum* and *D. radiodurans* were incubated at 30 °C for 48 h. For each culture, a 1 mL aliquot was plated onto nutrient agar plates in quadruplicate. Plates were incubated at 37 °C for 24 h (*E. coli*) and 30 °C for 48 h (*P. fungorum* and *D. radiodurans*). A 1 mL volume of sterile nutrient broth was used to flood the plate and a sterile scraper was used to remove the bacteria lawn from the agar. This was repeated with each seeded agar plate, for each biological repeat of each test sample. Each cell suspension was aseptically transferred to a sterile collection tube containing 20 mL of nutrient broth for incubation at the respective times and temperatures for each strain. Following incubation, tubes were vortexed for 10 s. The cultures were diluted in sterile peptone water (Sigma-Aldrich; 70179) to an OD600 nm of 1.0, prior to experimentation.

Peptone water was used to dilute the freeze thaw treatment samples to provide the test microbes with a minimal nutrient source to help support growth, which is best representative of what would be available on Mars, while 0.9% saline was used to dilute the standalone UV treatment samples as it is an isotonic solution and non-pigmented. This ensures full absorption of the UVC rays.

### 2.2. Freeze-Thaw Treatment

A Forma Scientific −86 °C Freezer (Thermo Fisher Scientific, Waltham, MA, USA) set to −80 °C with a temperature fluctuation ±0.5 °C, and a Julabo SW22 water bath set to 30 °C (monitored by mercury thermometer) was used for the freeze-thaw treatments of the test microbes. For each freeze-thaw cycle, ambient room temperature was 23 °C ± 2 °C. Aliquots of 1 mL of the standardized test sample (standardized to an OD600 nm of 1.0 as described above) were aseptically transferred into 20 sterile 1.5 mL microcentrifuge tubes and placed at −80 °C. Sample 1 was maintained at −80 °C until ready for analysis. After freezing for 24 h, samples 2−20 were removed from the −80 °C freezer and placed on ice (approx. 1.5–2.0 Kg), within a Cole Palmer ThermoSafe compact insulated shipper foam box (Fisher Scientific; 11775509). The box was then placed in a water bath set to 30 °C to allow a gradual thaw over an 8 h period until samples had melted under ambient room temperature conditions. Following this, samples 2–20 were placed directly into the water bath set at 30 °C for approximately 20 min allowing samples to reach 30 °C to facilitate reaching the average maximum surface temperatures experienced on Mars across one solar day during the summer at the equator [3]. Samples 2–20 were re-frozen at −80 °C for a further 16 h, to mimic expected temperatures on the surface of Mars when not exposed to the heat of the sun. Sample 2 was set aside at −80 °C. This concluded one full freeze-thaw cycle. Freeze-thaw cycles were repeated until all samples had been cycled for the indicated number of times.

Samples to be plated were removed from the freezer and allowed to thaw completely at ambient room temperature. They were then placed directly in the waterbath for 20 min to allow the temperature to reach 30 °C. A 100 μL aliquot of each sample was then subjected to a 1:10 serial dilution (100 μL sample + 900 μL sterile Ringer’s (96724-100TAB; Sigma-Aldrich)) and were aseptically plated on nutrient agar via standard spread plate method. Both *E. coli* strains were incubated at 37 °C for 18 h while *P. fungorum* and *D. radiodurans* were incubated at 30 °C for 48 h; all colony forming unit counts were then enumerated. The remaining 900 μL of test sample was subjected to UV dose response testing during the combination testing phase as detailed in Section 2.4 below.

**Figure 1:**
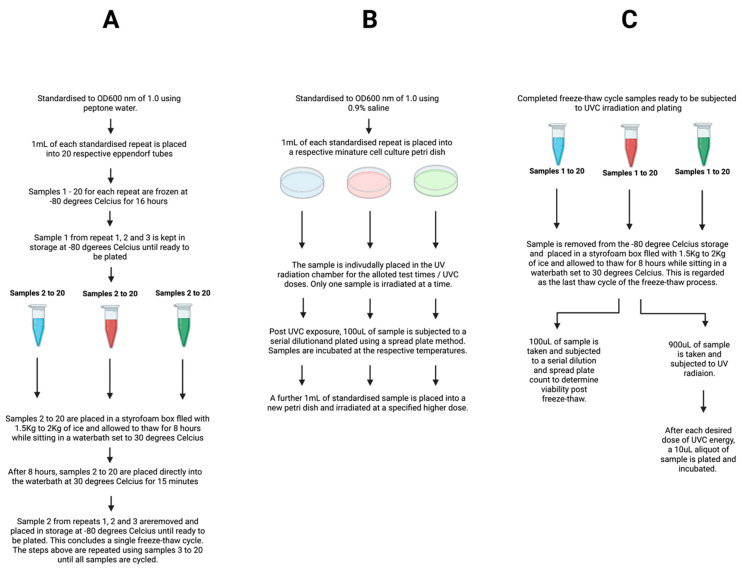
Schematic of the experimental workflow for the freeze-thaw process (**A**), the UVC radiation process (**B**) and the combined freeze-thaw and UVC radiation process (**C**).

The average CFU/mL counts for each freeze thaw cycle were normalized to the zero-time point in order to express average CFU/mL counts as a percentage of total viability, where 100% viability was based on the CFU/mL counts obtained for the zero-time point sample. See Figure 1A for a schematic overview of the freeze-thaw process.

### 2.3. UV Treatment

A CAMAG Cross-linker, housing a 254 nm 8 Watt, 288 mm long UV bulb (Labortechnik, Wasserburg, Germany) was used as the UV source. Standalone UV irradiation tests were conducted to determine the effect of UV irradiation on the test isolates with and without the second variable of freeze-thawing. As per previous studies, a 254 nm bulb was chosen as this wavelength is closest to the peak of DNA absorption and falls within the Martian UVC wavelength spectrum [23]. Prior to exposure, the 254 nm bulb was equilibrated using an optical power meter (Thorlabs: PM100D with a 0.7 cm^2^ aperture S120VC head) to determine the energy output in order to calculate the UV dose in J/m^2^. The dose (J/m^2^) was calculated as follows: Energy per m^2^ [J/m^2^] = Power per m^2^ [W/m^2^] × Exposure Time [s]. Power was determined by dividing the measured wattage reading from the UV energy detector by the energy detectors surface area (0.7 cm^2^). The CAMAG Cross-linker sample exposure area was mapped prior to experimentation using the optical power meter to determine the UV energy focal point. This area was marked, and test samples were positioned at this point to ensure consistency. This was performed for each experimental repeat to ensure an equal dose was delivered for all replicates. Prior to use, the CAMAG Cross-linker was sterilized with 70% Isopropyl-alcohol. A 0.9% saline solution was used to standardize prepared cells to a concentration of an OD600 nm of 1.0. A 1 mL volume of the biological replicate test samples (1, 2 and 3) was placed into sterile mini-tissue-culture plates (Sarstedt; 83.39) and swirled gently to ensure an even surface distribution. Samples were irradiated singly. For samples with long exposure times, the sample dishes were swirled at 10 min intervals and 45 µL of 0.9% saline was added to account for the loss in sample volume due to evaporation (the rate of which had been previously calculated; data not shown). Once the desired dose of UVC was delivered, the samples were mixed gently, and 1 mL was placed into a sterile 1.5 mL tube. A volume of 100 μL was taken from the tube and plated onto sterile nutrient agar plates using the standard spread plate method. A limited number of low-dose-exposure samples required 1:10 serial dilutions using sterile Ringers solution, to aid in enumeration, when the colony forming unit (CFU) counts were too numerous to count. Once the sample had been exposed to the first dose of energy and plated, a further 1 mL volume of sample was added to a new mini-tissue-culture plate (Sarstedt; 83.39) and exposed to a greater dose of energy until completed and plated as described. This was repeated until each dose of energy had been administered. Both *E. coli* strains were incubated at 37 °C for 18 h, while *P. fungorum* and *D. radiodurans* were incubated at 30 °C for 48 h; all were then enumerated. It must be noted that only one replicate was prepared and irradiated at a time. See Figure 1B for a schematic overview of the standalone UV radiation process.

### 2.4. Freezing Treatment Combined with UV Treatment

The remaining 900 μL of the initial freeze-thaw test sample (detailed in Section 2.2 above) was transferred to a sterile mini-tissue-culture plate and swirled gently to ensure an even surface distribution and irradiated in the same manner as described in Section 2.3. Samples with long exposure times were swirled at 10 min intervals and 45 µL of peptone water was added to account for the loss in sample volume due to evaporation. The samples were exposed to different doses of UVC based on the observed UVC sensitivity informed by earlier standalone UV testing. At each interval the samples were swirled and 10 μL of the test sample was taken from the petri dish and placed directly onto nutrient agar and spread using standard spread plate method. A limited number of low-dose-exposure samples required 1:10 serial dilutions (10 μL sample, 90 μL sterile Ringers) when the colony forming unit counts were too numerous to count for earlier timepoints. Both *E. coli* strains were incubated at 37 °C for 18 h, while *P. fungorum* and *D. radiodurans* were incubated at 30 °C for 48 h and then enumerated. See Figure 1C for a schematic overview of the combination freeze-thaw: UV process.

### 2.5. Data Analysis 

GraphPad Prism (Version 8.3.0) software was used to analyze the data, which were represented in the form of colony forming unit (CFU) counts per aliquot measurement of sample recorded, whether for freeze-thaw cycle and/or UV treatment. Due to the large numbers of cells recorded, CFU/mL values were converted into a Log10 format. Initial starting CFU/mL concentrations were recorded for each biological repeat, for each test sample across each stage of experimentation and taking those values to be representative of 100% viability for each biological replicate. Standard deviation was depicted as +/− one standard deviation about the mean of the triplicate assays conducted for each test sample. A repeated measures analysis of variance (ANOVA) was conducted to investigate whether there was a bacterium, freeze-thaw cycle and/or UV energy effect on viability, whereby viability was the measure, freeze-thaw cycles and UVC energy were the within-subject factors and bacterium was the between subject factors. The assumptions of normality of the standardized residuals and sphericity were investigated and analysis was performed accordingly. These statistical analyses were all performed in SPSS Version 27.

## 3. Results

### 3.1. The Effect of Freeze-Thawing on Bacterial Viability 

**Figure 2:**
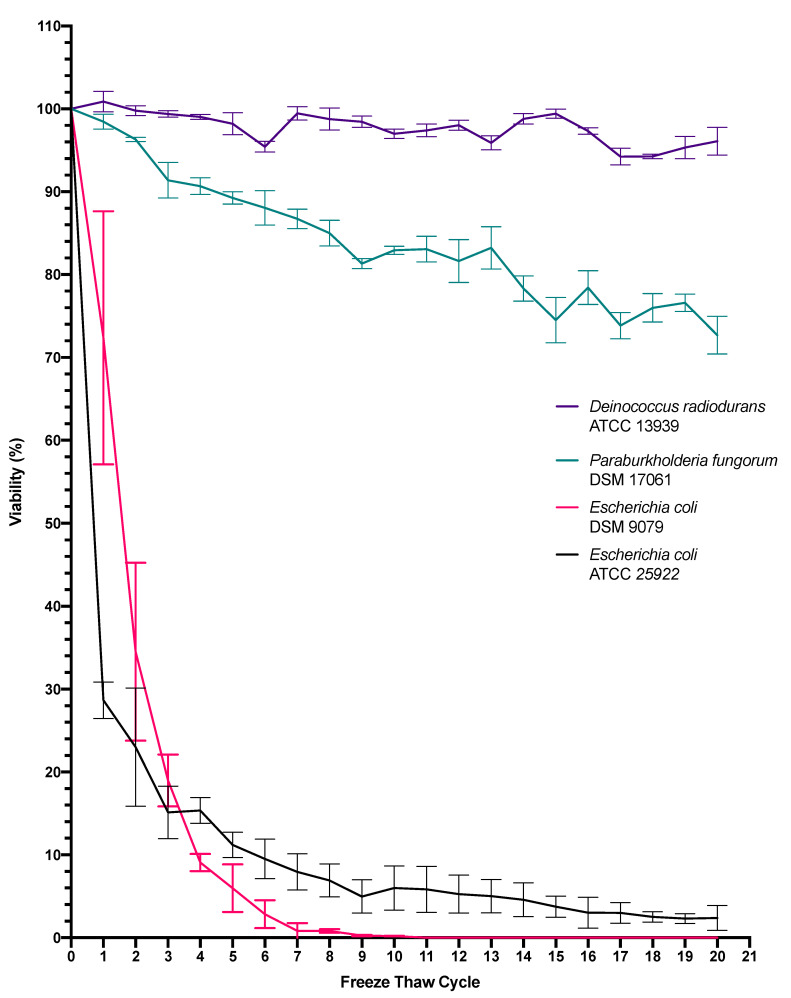
Percentage viability of *E. coli* ATCC 25922, *E. coli* DSM 9079, *P. fungorum* DSM 17061, *D. radiodurans* ATCC 1393 following successive freeze-thaw cycles (−80 °C to 30 °C). Note: Error bars depict +/− one standard deviation about the mean for each test sample.

In order to determine the viability of the selected test microbes when exposed solely to simulated Martian surface temperatures, successive freeze-thaw cycles were conducted in triplicate. Figure 2 shows the results of subjecting the four test strains to successive rounds of freeze-thawing. When each strain was standardized to an OD600 nm of 1.0, the zero-timepoint (T0) standardized starting concentrations were 7.42 × 10^8^ CFU/mL (*E. coli* ATCC 25922), 1.84 × 10^8^ CFU/mL (*E. coli* DSM 9079), 3.28 × 10^8^ CFU/mL (*P. fungorum* DSM 17061) and 3.92 × 10^7^ CFU/mL (*D. radiodurans* ATCC 1393). The error bars depict +/- one standard deviation about the mean for each test sample. Notably, the percentage viability of *E. coli* 25922 was reduced by 71.3% after a single freeze-thaw cycle, while 10% viability was reached between freeze-thaw cycles 5 and 6. In contrast, the viability of *E. coli* DSM 9079 decreased by 50 % between freeze-thaw cycle 1 and 2, while 10% viability was observed between cycle 3 and 4. The percentage viability of *P. fungorum* DSM 17061 remained at 72.6% following a 20-freeze-thaw cycle period, while *D. radiodurans* ATCC 13939 exhibited a viability of 96% at the end of 20 successive rounds of freezing and thawing.

Meanwhile, statistical analysis showed a difference between both *E. coli* strains with respect to themselves and the other test strains. Further analysis of *E. coli* ATCC 25922 showed a significant difference between freeze-thaw cycle 0 and freeze-thaw cycle 4 (*p* = 0.002), and again between cycle 8 and cycle 12 (*p* = 0.018), however, thereafter no significance was recorded in between cycles 8 to 12 (*p* = 0.304), 12 to 16 (*p* = 0.312) and 16 to 20 (*p* = 0.098). Statistical analysis of *E. coli* DSM 9079 showed significance between freeze-thaw cycles 0 to 4 (*p* = 0.0005), yet no significance from cycle 4 to 8 (*p* = 0.053), 8 to 12 (*p* = 0.204), 12 to 16 (*p* = 0.216) and 16 to 20 (*p* = 0.216). When subjected to statistical analysis, *P. fungorum* was found to be significantly different to each of the other test strains, yet no significance was recorded between freeze-thaw cycles 0 to 4 (*p* = 0.064) and 4 to 8 (*p* = 0.137), 8 to 12 (*p* = 0.250), 12 to 16 (*p* = 0.315) and 16 to 20 (*p* = 0.115). However, significance was seen when cycles 0 to 10 (*p* = 0.004) and 10 to 20 (*p* = 0.015) were statistically analyzed, thus showing a freeze-thaw effect over a longer range of time in comparison to the *E. coli* strains, which were more sensitive to the effects of freeze-thaw.

Statistical analysis, *D. radiodurans* was found to be significantly different to each of the other test strains, yet no significance was recorded between freeze-thaw cycles 0 to 4 (*p* = 0.505), 4 to 8 (*p* = 0.809), 8 to 12 (*p* = 1.0), 12 to 16 (*p* = 0.862) and 16 to 20 (*p* = 0.718). Again, significance was not seen when cycles 0 to 10 (*p* ≥ 0.05) and 10 to 20 (*p* ≥ 0.05) were statistically analyzed, thus allowing for the conclusion that over the course of 20 freeze-thaw cycles, there is no long-term freeze-thaw effect seen on *D. radiodurans*.

### 3.2. Dose Response of Bacterial Viability following UVC Exposure

**Figure 3:**
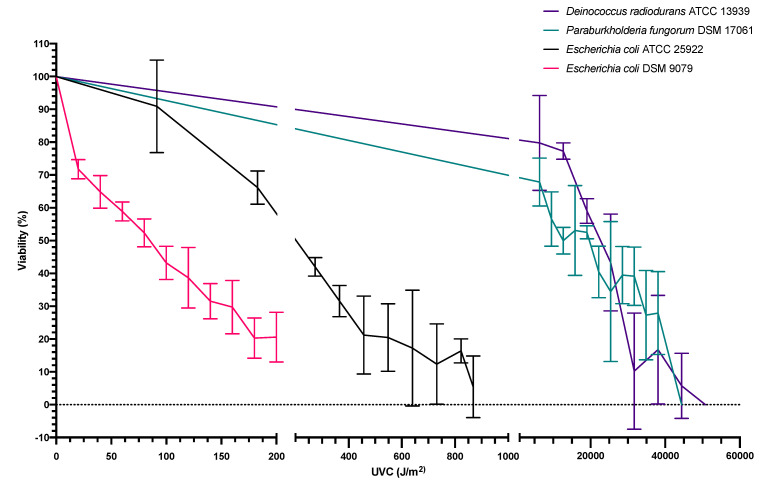
Percentage viability of *E. coli* ATCC 25922, *E. coli* DSM 9079, *P. fungorum* DSM 17061, *D. radiodurans* ATCC 1393 following exposure to a range of intervals of UVC doses, spanning from 0–870 J/m^2^, 0–200 J/m^2^, 0–44,415 J/m^2^ and 0–50,760 J/m^2^ of UVC irradiation, respectively. The error bars depict +/− one standard deviation about the mean for each test sample.

In order to determine the viability of the selected test microbes when exposed solely to simulated Martian UVC irradiation, independent UVC-dose-response-experimentation at 254 nm was performed. Treatments were conducted in triplicate, and the average CFU/mL counts of the three zero-time point test samples formed the basis for the average starting concentrations. When each strain was standardized to an OD600 nm of 1.0, the T0 standardized starting concentrations were 8.36 × 10^7^ CFU/mL (*E. coli* ATCC 25922), 1.88 × 10^8^ CFU/mL (*E. coli* DSM 9079), 5.1 × 10^8^ CFU/mL (*P. fungorum* DSM 17061) and 5.03 × 10^7^ CFU/mL (*D. radiodurans* ATCC 1393). Each test sample was standardized to an OD600 nm reading of 1.0. The error bars depict +/− one standard deviation about the mean for each test sample. Experimentation was not conducted in monolayers in order to simulate how the microbial colonies would be found/behave on Mars if grown in biofilm-forming colonies on the surface or within bioreactor-like vessels. Figure 3 shows the results of subjecting *E. coli* ATCC 25922 to independent UV treatment, with each point on the graph representing the average value of triplicate testing. Consequentially, the percentage viability of *E. coli* ATCC 25922 was reduced by 50% when exposed to 245 J/m^2^ of UVC irradiation. As expected, *E. coli* DSM 9079 exhibited even greater sensitivity to UVC irradiation with a 50% reduction in viability observed at a dose of 85 J/m^2^. When *P. fungorum* DSM 17061 was subjected to independent UV treatment the percentage viability of the sample was reduced by 50 % when exposed to 12,715 J/m^2^ of UVC irradiation. As depicted in Figure 3, when *D. radiodurans* ATCC 13939 was exposed to independent UV treatment, the percentage viability of *Deinococcus radiodurans* ATCC 13939 was reduced by 50% upon exposure to 22,653 J/m^2^ of UVC irradiation.

### 3.3. Bacterial Viability following Dual Exposure to Freeze-Thawing and UVC Radiation 

Once survival parameters were determined for the individual treatments, the viability of the selected test microbes when exposed to a simulated combination of both Martian equatorial surface temperatures and UVC irradiation at 254 nm was determined. Successive freeze-thaw cycles and UVC dose response experiments were conducted in triplicate, using samples obtained from the individual freeze-thaw treatments. The average CFU/mL counts of the three zero-time point test samples formed the basis for the average starting concentrations.

**Figure 4:**
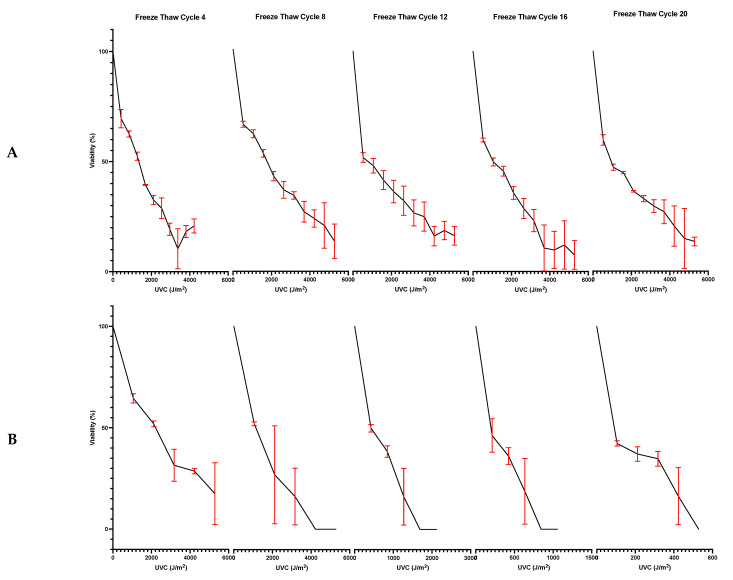

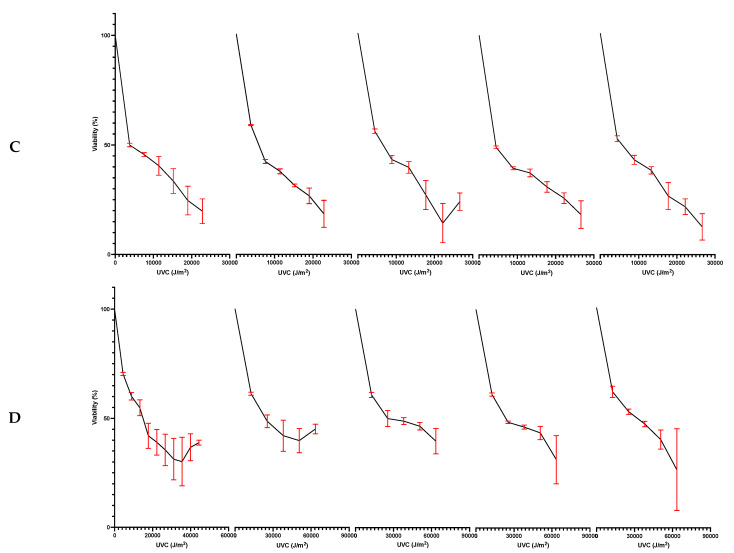
(**A**) Viability of *E. coli* ATCC 25922 over successive freeze-thaw cycles from an average starting concentration of 7.42 × 10^8^ CFU/mL when subjected to UVC irradiation ranging from 0–5285 J/m^2^. (**B**) Viability of *E. coli* DSM 9079 over successive freeze-thaw cycles from an average starting concentration of 1.84 × 10^8^ CFU/mL when subjected to UVC irradiation ranging from 0–5285 J/m^2^. (**C**) Viability of *P. fungorum* DSM 17061 over successive freeze-thaw cycles from an average starting concentration of 3.28 × 10^8^ CFU/mL when subjected to UVC irradiation ranging from 0–25,371 J/m^2^. The error bars depict +/− one standard deviation about the mean for each test sample. (**D**) Viability of *D. radiodurans* ATCC 13939 over successive freeze-thaw cycles from an average starting concentration of 3.92 × 10^7^ CFU/mL when subjected to UVC irradiation ranging from 0–63,428 J/m^2^.

Figure 4A shows the results of subjecting *E. coli* ATCC 25922 to successive rounds of freeze-thawing each followed by UV treatment. *E. coli* ATCC 25922 is a well-documented species of *E. coli* and has an intact SOS response system. This defined the parameters of survival when looking at the *RecA-* strain of *E. coli.* At freeze-thaw cycle 4, 8, 12, 16 and 20 (which were chosen as even intervals to denote a change over time), the percentage viability of *E. coli* ATCC 25922 was reduced by 50% at approximately 1270 J/m^2^, 1580 J/m^2^, 1060 J/m^2^, 1060 J/m^2^ and 1000 J/m^2^, respectively. Figure 4B illustrates *E. coli* DSM 9079 when exposed to successive rounds of freeze-thawing followed by UV treatment. At freeze-thaw cycle 4, 8, 12, 16 and 20, the percentage viability of *E. coli* DSM 9079 was reduced by 50% at approximately 2200 J/m^2^, 1060 J/m^2^, 430 J/m^2^, 215 J/m^2^ and 125 J/m^2^, respectively. Figure 4C depicts *D. radiodurans* ATCC 13939 when subjected to successive rounds of freeze-thawing followed by UV treatment. As a true extremophile, it was expected to be the most resistant to Mars-like conditions of all the test microbes, thus setting the limits for UVC exposure. At freeze-thaw cycle 4, 8, 12, 16 and 20, the percentage viability of *D. radiodurans* ATCC 13939 was reduced by 50% at approximately 16,150 J/m^2^, 24,500 J/m^2^, 25,370 J/m^2^, 24,000 J/m^2^ and 23,500 J/m^2^, respectively. Figure 4D shows the results of subjecting *P. fungorum* DSM 17061 to successive rounds of freeze-thawing followed by UV treatment. At freeze-thaw cycle 4, 8, 12, 16 and 20, the percentage viability of *P. fungorum* DSM 17061 was reduced by 50% at approximately 3800 J/m^2^, 5850 J/m^2^, 5000 J/m^2^, 4500 J/m^2^ and 4300 J/m^2^, respectively.

## 4. Discussion

### 4.1. Freeze-Thaw Cycle Exposure

The effects of Mars-like environmental conditions (freeze-thaw and UVC radiation) on bioremedial microbial strains was investigated to determine the limits that microbial life can withstand in a Mars-like environment. It is known that freezing and thawing impose several interconnected stresses on cells, including dehydration, hyperosmotic stress, ice formation and oxidative stress. In addition to this, other variables such as nutrient availability and the rate of cooling all play a role in cellular viability within a freeze-thaw environment [24,25,26]. Taking this into account, and average daily temperatures on equatorial Mars cycling from −63 °C to 30 °C [3], the effect of freeze-thaw cycles on the chosen bacterial strains was investigated in order to determine the survivability of selected bacterial strains within a minimal nutrient-Mars-like environment over time and to produce a baseline study, from which to inform future functional studies.

Figure 2 illustrates the percentage reduction in viability of selected bacterial strains when subjected to the daily temperature fluctuations experienced on the equatorial surface of Mars. Martian surface temperatures range from −140 °C to +30 °C, with a daily average of −63 °C [3]. The experimental design encompassed a range of temperatures spanning −80 °C to +30 °C, incorporating the variability expressed in natural ambient atmospheric changes such as ambient temperature. The results presented in Figure 2 demonstrates that *E. coli* ATCC 25922 can withstand at least 20 successive freeze thaw cycles. While an average reduction in viability of 71.3% was observed between cycle 0 and cycle 1, the remaining 28.7% of the population were able to withstand successive cycles in the absence of a cryoprotectant with a gradual decline in viability being observed over time. While it is known that the formation of intracellular ice crystals can cause cells to lyse completely, there is also a molecular mechanism to this process [27]. As demonstrated by Alur and Grecz (1975), the reduction in bacterial viability when exposed to freezing and thawing conditions is proposed to be largely caused by single strand DNA breaks, and consequential DNA degradation [28]. It should be noted that it is not possible to form a direct comparison between the work of Alur and Grecz, and that of the work within this study due to differences in experimental parameters; for example, a different strain of *E. coli* (*E. coli* B/r), in the absence of a cryoprotectant, was used in the 1975 study and only a single freeze-thaw cycle (unlike this study where 20 freeze-thaw cycles were examined) was observed with temperatures ranging from −196 °C to +25 °C. Similarly, a study conducted by Lyscov based on the intrinsic viscosity and sedimentation constants of calf-thymus DNA and phage-T2 DNA, showed that a decrease in molecular weights of the DNA samples was caused by freeze-thaw, while another study by Shao determined that progressive DNA degradation of blood samples was observed in samples with DNA sizes larger than 100 kb, as these were found to be most sensitive to freeze/thaw degradation. It was also seen that increasing the DNA concentration of the stored samples from 10 μg/mL to 100 μg/mL had a protective effect on DNA stability [29,30].

When examining the percentage viability of *E. coli* DSM 9079, as seen in Figure 2, *E. coli* DSM 9079 acted as the SOS-repair-system-deficient control within this study. *E. coli* DSM 9079 was a *ΔrecA* mutant, while *E. coli* ATCC 25922 had the functional *RecA* gene. *RecA* encodes a multifunctional enzyme that plays an important role in DNA repair, homologous recombination and the induction of the SOS biomolecular response systems within the cell [13]. While not genetically identical to *E. coli* ATCC 25922, over the course of the successive freeze-thaw cycles, it is evident that absence of the *RecA* gene played an important role in the survivability of *E. coli* DSM 9079 in response to freeze thawing. When compared to the more robust control (*E. coli* ATCC 25922), which survived 20 successive freeze-thaw cycles, the *ΔrecA* mutant was non-viable by freeze-thaw cycle 9. Statistical analysis determined that there were differences amongst both *E. coli* strains when subjected to repeated freeze-cycles, as stated in the results. While genomic studies are indicated, it could be hypothesized that DNA repair systems are activated after this primary freeze-thaw cycle, owing to the prolonged survival of the remaining 28.7% of *E. coli* ATCC 25922 cells (yet it should be mentioned that there is no published literature to support this hypothesis and, as such, a follow up study would be required).

Conversely, it has been shown in another study that *E. coli* B cell-line progeny cells, which had been exposed to daily freeze-thaw cycles, developed on average a 54% mortality rate to freeze-thaw conditions compared to their less sensitive ancestors that showed an average mortality rate of 34% [31]. It is noted that between cycle 0 and cycle 1, *E. coli* DSM 9079, unexpectedly, exhibited an average initial decrease in viability of 28%, whereas *E. coli* ATCC 25922 had an average initial decrease of 71.3%, although was recoverable in small numbers after 20 cycles.

When *P. fungorum* DSM 17061 was exposed to the same set of freeze-thaw conditions as both the robust and sensitive controls, it exhibited an increased resistance to the effects of freeze-thaw. Indeed, a negligible average initial decrease in viability between cycle 0 and cycle 1 of 2% was observed, and over the course of 20 cycles, the 50% viability threshold had not been reached. This strain exhibited an average viability of 76%; meaning it is capable of withstanding successive freeze-thaw cycles without a dramatic loss in cell population when beginning with a starting concentration of 3.28 × 10^8^ CFU/mL. Based on these data, it could be suggested that due to the relatively large genome size of *P. fungorum* DSM 17061 (8.1 Mb in contrast to *E. coli* ATCC 25922′s genome, which is 5.2 Mb [32,33]), there may be genes encoding for both different and more efficient repair systems; such as the *UvrB, RuvB* and *RecG* genes observed in the DNA repair of *Burkholderia cepacia* when subjected to the toxic effects of trichloroethylene in mutagenic studies [34]. In addition, the large genome size of *P. fungorum* may be a contributing factor to the bacterium’s tolerance of successive freeze-thaw cycles and its ability to prolong survival as detailed earlier by Shao [30].

*D. radiodurans* ATCC 13939 is a well-documented bacterium in the field of astrobiology, largely due to its renowned radio-resistance. It has been shown that *D. radiodurans* uses redundant ‘multi-genomic’ copies to aid in DNA repair by means of inter-chromosomal recombination [18,35], and this ensures genomic stability as the process is highly specific and not prone to error. As shown in Figure 2, the influence of freeze-thaw and the succedent cycles appear to have minimal effects on the longevity and viability of the triplicate test samples. Indeed, between cycle 0 and cycle 1, the initial viability was 100%. Like *P. fungorum* DSM 17061, the 50% viability threshold was not reached over the course of the 20 cycles. Remarkably, after the last experimental cycle (cycle 20) the average cell viability was 96%. When the data are compared to the findings of Alur and Grecz (1975), where *E. coli* B/r was subjected to a single freeze-thaw cycle with a temperature range of −196 °C to 25 °C (which concluded that freeze-thaw causes DNA degradation), the data recorded here further supports their research due to the known efficiency of *D. radiodurans* DNA repair systems, and its evidential ability to survive successive freeze-thaw cycles. If multiple genomes and highly efficient DNA repair systems are key in surviving the effects of freeze-thaw, it could be suggested that *P. fungorum* DSM 17061′s single yet large genome, may encode repair systems that are more efficient than the robust control *E. coli* strain, although less efficient than *D. radiodurans* multiple genomes. Statistical analysis helps to further support the hypothesis that *P. fungorum* is just as resistant to external environmental insults as *D. radiodurans*, as will be discussed further on in this paper.

While examining the effects of freeze-thaw, it is important to highlight the importance of the work carried out in furthering our understanding of how these microbes react to extra-terrestrial conditions, as there is no available literature focused solely on the long-term independent freeze-thaw cycling of *E. coli* ATCC strain 25922, *E. coli* DSM strain 9079, *P. fungorum* DSM strain 17061 and *D. radiodurans* ATCC strain 13939. Recorded bacterial strains exposed to freeze-thaw experimentation within the published literature include *E. coli* B [31]. It was found that ancestral cells, when exposed to daily freeze-thaw cycles were more tolerant of the temperature cycles in comparison to their generational progeny. When compared to the results of this study, it is clear that continual freezing and thawing, even in progeny cells, weakens cells and reduces viability under these conditions. It is noted that an initial decrease in viability of 35.2% between freeze-thaw cycle 0 and 1 was observed in ancestral cells, in comparison to the 71.3% initial reduction seen in this study. This may indicate that different strains of *E. coli* can tolerate freeze-thaw to varying degrees, however it must be stated, that in this study, cells were slowly thawed over 8 h and heated to 30 °C, whereas Sleight et al., thawed at room temperature (22 °C) for 1.5 h, thus not allowing for direct comparisons. Interestingly, when *Pseudomonas paucimobilis* [36] was exposed to an initial freeze-thaw, a 40–60% decrease in bacterial population was observed, with sustained levels thereafter. When compared to the experiments carried out in this study, a similar finding was reported, showing a large initial drop in viability, as seen with the *E. coli* strains, with sustained levels thereafter, and also evident with *D. radiodurans* and *P. fungorum.*

### 4.2. Molecular Influences Associated with Freeze-Thaw Cycle Exposure

While examining the effects of freeze-thaw on both *E. coli* strains, it is possible that the lack of a DNA repair system within *E. coli* DSM 9079 may upregulate another response pathway attributing to an initial reduction in freeze-thaw mediated cellular damage during the first freeze-thaw cycle, such as the induction of a nucleotide excision repair system consisting of the genes *uvrA*, *uvrB*, *uvrC* and *uvrD* [37]; in comparison to *E. coli* ATCC 25922, which experienced a dramatic initial decrease based on the action of a competent cell regulatory system in response to damage, yet sustained numbers throughout unlike *E. coli* DSM 9079. One study notes that low temperature and long storage time could induce the incomplete disassembly of formed *RecA*-DNA nucleoprotein filaments, thus causing a failure in cell repair in cells with fully functioning repair systems [38], which may explain the differences observed in the initial decrease in cell viability between the two strains.

While hypothesizing why *P. fungorum* has shown itself to be equally resistant to environmental insults as *D. radiodurans*, this may be due to DNA compaction. It is important to note, the nucleoid of radioresistant microbes, such as *D. radiodurans*, are known to be highly condensed structures, which allow the organism to withstand high doses of irradiation (DNA damage) while remaining unaltered [39]. This is due to the presence of the HU protein, which plays a crucial part in the organization and compaction of DNA in *D. radiodurans*. *P. fungorum* was also determined to be a highly UV resistant microbe, and while there is no published literature to support the existence of HU protein in *P. fungorum*, it has been shown that *Paraburkholderia cenocepacia* (a closely related strain) expresses HU protein [40]. Therefore, DNA compaction found within the nucleoid of both *P. fungorum* and *D. radiodurans* may facilitate a tolerance to DNA degradation caused by freeze-thaw conditions.

Microbial molecular adaptations to the cold include slow chemical reaction rates and limited enzyme activity, denaturation of proteins, increased water viscosity, decreased cell membrane fluidity and limited water availability for biochemical reactions [41,42,43,44,45], the production of cold-shock proteins, altered metabolisms and homeoviscous adaptation (the alteration of the cell membrane composition to maintain cell membrane fluidity in response to environmental changes) [45,46]. During heat-/cold-shock, cell membrane fluidity and enzyme activity are decreased, resulting in the reduced efficacy of transcription and translation due to the stabilization of nucleic acid secondary structures. In addition, protein folding and ribosome function is compromised [47]. Cold-shock proteins serve as nucleic acid chaperones that may prevent the formation of secondary structures in mRNA at low temperatures, thus combatting the harmful effects of low temperatures. While cold-shock proteins have not yet been experimentally isolated within *E. coli* ATCC 25922, there are multiple studies demonstrating the production of heat-shock proteins in response to temperatures of 45 °C and above [48,49]. While heat- and cold-shock proteins are not encoded for by the same genes, the *E. coli* genus is known to be a carrier of cold-shock proteins [50], therefore with the known production of heat-shock proteins in *E. coli* ATCC 25922, there is a strong possibility that cold-shock proteins are also playing a role in the bacterium’s prolonged survival. In both proof of concept, and in demonstration of the converse, *RecA* has been implicated as a cold-shock inducing protein [51]. *E. coli* DSM 9079 (a *ΔrecA* mutant) exhibits reduced viability in response to freeze-thaw cycling. Again, while not directly comparable due to the speciation differences, it is possible that cold-shock proteins, or their absence, could play a role in the extent of the observed viability. Previous studies have shown that *D. radiodurans* is capable of producing both heat-shock and cold-shock proteins. One study found an increased expression of 67 proteins during heat-shock and 42 proteins during cold-shock (observed by two-dimensional polyacrylamide gel electrophoresis (2D PAGE) and autoradiography) [52]. It must be noted, that within the study “cold-shock” is denoted by a cooling stage of 20 °C, and not sub-zero temperatures. A recent meta genomic analysis of the *P. fungorum* genome has also revealed the presence of cold-shock proteins [53]. When comparing the available literature to the results seen in this study, it is probable that cold-shock proteins have a role to play in the adapted and prolonged survival of these microbes to the effects of freeze-thaw. 

While not greatly studied in a diverse range of prokaryotic organisms, the process of homeoviscous adaptation may also play a role in the prolonged survival of these microbes. Microbes that are capable of synthesizing their own fatty acids include *E. coli*, *Paraburkholderia*, and *Deinococcus* [54,55,56]. These fatty acids have diverse melting points in order to remain in the necessary physical form depending on the temperature of the surrounding environment. An increase in the amount of low-melting-point fatty acids (such as monounsaturated fatty acids, polyunsaturated fatty acids and branched-chain fatty acids relative to their saturated straight analogues) provides adequate membrane fluidity in almost all organisms [45,57,58,59]. It has been shown experimentally how psychrotolerant bacteria (*Pseudomonas*, *Arthrobacter*, *Flavobacterium* and *Deinococcus*) were able to change their lipid membrane structures in order to retain fluidity depending on the temperature. It was found that this fatty acid distribution was also affected significantly by the growth temperature of the surrounding environment. It is noted that there is no testing completed to date on the effects of membrane lipid distribution during freeze-thaw and UVC irradiation.

### 4.3. UV Exposure

The effect of independent exposure to damaging UVC irradiation was investigated to observe how these bacterial strains behave when exposed to UVC irradiation similar to the Martian environment (200–280 nm). The results were intended to inform future studies into the suitability and longevity of bacteria within extra-terrestrial habitats when exposed to UVC irradiation at 254 nm.

Figure 3 illustrates the percentage reduction in viability of these bacterial strains when subjected to increasing doses of UVC energy. A 50% reduction in viability was observed when *E. coli* ATCC 25922 was exposed to 245 J/m^2^, resulting in a standard dose response curve. When compared to *E. coli* DSM 9079, over four times the amount of UVC energy was needed to inactivate *E. coli* ATCC 25922 compared to the *ΔrecA* mutant *E. coli* DSM 9079, which required a UVC dose of 85 J/m^2^ in order produce a 50% reduction in viability. Again, while not genetically identical, this finding reaffirms the important function of the *RecA* gene in DNA repair/homologous recombination and the SOS biomolecular response systems. These systems are needed as UVC energy is absorbed by DNA causing the dimerization of nucleic acid bases including cyclobutane pyrimidine species and pyrimidine (6–4) pyrimidone compounds, together with their Dewar isomers [6]. When compared to the *RecA+* strain (*E. coli* ATCC 25922), they denote a “sensitive” and “competent SOS response” range for judging the viability of the test strains when exposed to simulated Mars-like conditions. They allow for the creation of a guideline of the lower and upper limits of survival for the average terrestrial microbe.

Figure 3 also illustrates the UVC resistance of *P. fungorum* DSM 17061. It was noted that, experimentally, 10,000 J/m^2^ of UVC energy was required to reduce the viability of *P. fungorum* DSM 17061 by 50 %, with 44,415 J/m^2^ of UVC energy being required to inactivate the sample completely. When the UVC resistance of *P. fungorum* DSM 17061 was compared to those of a known radio-resistant bacterial strain (*D. radiodurans* ATCC 13939), 25,000 J/m^2^ of UVC energy was required to reduce viability by 50 %, with complete inactivation of the test sample observed at 50,760 J/m^2^. When the death points of both species were compared, a difference of 6345 J/m^2^ was recorded, suggesting that *P. fungorum* DSM 17061 is a highly UV-resistant micro-organism, which has not been reported before.

When subjected to statistical analysis, a significant difference was not seen in the recorded viabilities of *P. fungorum* and *D. radiodurans* in response to comparable UVC energy doses (*p* = 0.685). The lack of significance denotes that while both species are not totally comparable, they are not statistically dissimilar to each other. This further supports the hypothesis that *P. fungorum* is akin to *D. radiodurans* in its ability to withstand extreme environmental insults. It is noted that a 254 nm wavelength was chosen for both its maximal absorbance by DNA and based upon its lack of photoreactive properties. Photoreactivation is the phenomenon by which UV-inactivated organisms regain their activity via photorepair of UV-induced lesions in the DNA by utilizing the energy of near-UV light (310–480 nm) and an enzyme, photolyase [60,61,62].

### 4.4. Molecular Influences Associated with UV Exposure

While photolyases allow for the mitigation of UV damage, most bacteria have either a single photolyase or photolyase-like gene with varying expression levels [63]. Studies examining DNA repair of *E. coli* at 254 nm based upon photoreactivation found that wavelengths from 220 nm to 300 nm reduced the level of photorepair in pyrimidine dimers due to a possible interference of the photolyase repair enzyme. As a result, the tests carried out in this paper were subjected to low levels of photorepair occurrences.

UV resistance is has also been linked to a high intracellular ratio of manganese (Mn) and iron (Fe). Interestingly, this phenomenon is proven to be irrespective of the surrounding levels of Mn and Fe within the environment [64]. *D. radiodurans* is one of the most radiation resistant life forms on the planet and as a result is often used as a positive control to demonstrate a high level of radiation resistance within an experimental study [65]. Interestingly, it is also known to contain high levels of intracellular Mn and Fe. This was found to be the result of the manganese efflux protein; MntE [66].

Based on these data, it is possible that an abundance of intracellular Mn and Fe may be contributing to the UVC resistance of *P. fungorum*, in addition to the possibility of DNA compaction as discussed earlier. Despite the paucity of research on this species, the identification of a type VI secretion system, T6SS-4, is implicated in the metal acquisition of intracellular manganese to protect from oxidative stress within the species *Paraburkholderia thailandensis* [67].

*E. coli* as a species does not routinely import Mn into the cell, although it has been shown to do so when iron is unavailable. This allows Mn to act as a substitute for iron, a common enzyme co-factor [68]. It does so using a small protein MntS and exporter MntP. The MntS protein is synthesized when manganese levels decline; it helps to enlarge the manganese pool. In contrast, MntP is a manganese exporter that is synthesized when intracellular manganese levels rise; preventing manganese levels from becoming too high and toxic [68]. Interestingly, it has been shown that *RecA* plays no part within the induction of Mn-superoxide-dismutase (which prevents the oxidative stress caused by high Mn levels) [69].

### 4.5. Combined Freeze-Thaw and UV Radiation

When the effects of the combined treatment of both freeze-thaw and UVC irradiation on the chosen bacterial strains was investigated, a species-dependent synergistic and antagonistic effect was observed. As mentioned, UVC damages active cells by inducing changes in the chemical structure of the DNA strands, resulting in the production of cyclobutane pyrimidine dimers. These dimers cause DNA molecule distortion, which in turns causes defects in DNA replication, which can lead to cell death [70,71]. In addition, freezing induces cellular senescence [31]. While species-dependent, the addition of UVC energy to freshly thawed cells may have less of an effect on DNA distortion as the cell is not fully active and is still recovering from being held in a stationary phase. In conjunction to the lack of DNA replication during stationary phase, it is possible that DNA becomes more compact, as demonstrated by the global DNA compaction in stationary phase by Janissen [72], thus protecting the DNA strands from the effects of UVC damage. Multiple studies carried out using *E. coli* strains and different bands of the UV spectrum consistently illustrate the effects of growth phase on UV resistance. It has been demonstrated how *E. coli* strains show a significant resistance to UV-induced damage when in stationary phase, as opposed to when exponentially growing during log phase [73,74]. As shown in Figure 4A,B this created an antagonistic effect in prolonging cellular viability, while Figure 4C demonstrates a synergistic effect and Figure 4D also demonstrates a synergistic effect as the combination of both factors aids in increasing cell death.

Figure 4A–D illustrate the percentage reduction in viability of these bacterial strains when subjected to the daily temperature fluctuations experienced on the equatorial surface of Mars (−140 °C to 30 °C with UV wavelengths of 200–400 nm). During the UV:Freeze-thaw experimentation, 900 μL aliquots of the sample were used and intermittently agitated, to avoid any possible shielding effect caused by having a large mass of bacterial cells; while 10 μL aliquots were cultured for colony counting in order to reduce experimental variation caused by a loss of cells.

Upon independent exposure to UVC energy, 200 J/m^2^ of energy was required to reduce the viability of *E. coli* ATCC 25922 by 50% (Figure 3). When these cells were subjected to a combination of both successive freeze-thaws and UV irradiation (Figure 4A), at freeze-thaw cycle 4, 8, 12, 16 and 20, the percentage viability of *E. coli* ATCC 25922 was reduced by 50% at approximately 1270 J/m^2^, 1580 J/m^2^, 1060 J/m^2^, 1060 J/m^2^ and 1000 J/m^2^, respectively. This shows a 6.4-, 7.9-, 5.3-, 5.3- and 5-fold increase in the amount of energy needed to reach the 50% viability threshold in comparison to the independent UVC tests, across each respective freeze-thaw cycle. Therefore, it can be said that freezing and thawing in the absence of a cryoprotectant extends bacterial cell longevity in the presence of a known cytotoxic dose of UV irradiation. This phenomenon was also reported by Gao and Leung (2015) where a significant reduction in UV inactivation was observed in cells that had been frozen prior to UVC irradiation (UV fluences of 15 to 90 J/m^2^ were used) with *E. coli* ATCC 25922 showing more UV resistance than the other test organism *E. coli* O157:H strain 961019 [15].

When *E. coli* DSM 9079 was independently exposed to UVC energy, a dose of 75 J/m^2^ was required to reduce viability by 50%. Similarly, when exposed to a combination of both successive freeze-thaws and UV irradiation (Figure 4B), at freeze-thaw cycle 4, 8, 12, 16 and 20, the percentage viability of *E. coli* DSM 9079 was reduced by 50% at approximately 2200 J/m^2^, 1060 J/m^2^, 430 J/m^2^, 215 J/m^2^ and 125 J/m^2^, respectively. This shows a 2.9-, 1.4-, 5.7-, 2.8- and 1.7-fold increase in the amount of energy needed to reach the 50% viability threshold in comparison to the independent UV tests, across each respective freeze-thaw cycle. Again, this demonstrates the importance of the *RecA* gene, when compared to the UV resistance of *E. coli* ATCC 25922, in supporting cell survival, and demonstrates how the *ΔrecA* deletion is detrimental to cell survival. Even so, given the enhanced UV sensitivity of the *ΔrecA* mutant, bacterial resistance to UV was still observed and survival was extended. It must be noted that all independent freeze-thaw data represent 10^−4^ CFU/mL counts of the test samples, whereas all combination testing represents counts ranging from neat to 10^−5^ CFU/mL dilutions. This accounts for the difference seen between the death of *E. coli* DSM 9079 by freeze-thaw cycle 9 during the independent testing, and its survival to upwards of 20 freeze-thaw cycles during the combination testing with UV and freeze-thawing.

During the independent freeze-thaw testing and combination testing, it was observed that despite being a *ΔrecA* mutant, *E. coli* DSM 9079 appeared to be more resistant to the initial effects of freeze-thaw when compared to *E. coli* ATCC 25922. While it is unknown if this delayed response phase could be strain-dependent or due to the influence of the *ΔrecA* mutation, *E. coli* ATCC 25922 ultimately survived its *ΔrecA*-deficient counterpart.

When *D. radiodurans* ATCC 13939 was initially exposed independently to UVC energy, a dose of 25,000 J/m^2^ was required to reduce viability by 50%. At freeze-thaw cycle 4, 8, 12, 16 and 20, the percentage viability of *D. radiodurans* ATCC 13939 was reduced by 50% at approximately 16,150 J/m^2^, 24,500 J/m^2^, 25,370 J/m^2^, 24,000 J/m^2^ and 23,500 J/m^2^, respectively. This denotes a 1.5-, 1.02-, 1.04- and 1.06-fold reduction in the amount of energy needed to reach the 50% viability threshold in comparison to the independent UV tests, across freeze-thaws 4, 8, 16 and 20, whereas a 1.01-fold increase in bacterial longevity was observed at cycle 12. It must be noted that these values are very close in range across all the freeze-thaw cycles, which shows that the combination testing had a minimal effect on bacterial viability, as is evident in Figure 4C. The increase in cell inactivation may be due to the possibility that for the multi-genome repair systems to operate, this strain must be actively in lag/log-phase, rather than a prolonged stationary phase, as seen by the levels of gene expression responsible for exponential growth and transient metal accumulation observed during lag/log phase in *Salmonella typhimurium* [75].

*P. fungorum* DSM 17061 also demonstrated a synergistic response to the combination of UVC and freeze-thaw, and became sensitive to the combined parameters, as seen in Figure 4D. Initially, when exposed independently to UVC energy, a dose of 10,000 J/m^2^ was required to reduce viability by 50%. At freeze-thaw cycle 4, 8, 12, 16 and 20, the percentage viability of *P. fungorum* DSM 17061 was reduced by 50% at approximately 3800 J/m^2^, 5850 J/m^2^, 5000 J/m^2^, 4500 J/m^2^ and 4300 J/m^2^, respectively. This demonstrates a 2.6-, 1.7-, 2.0-, 2.2- and 2.3-fold reduction in the amount of energy needed to reach the 50% viability threshold in comparison to the independent UV tests, across each respective freeze-thaw cycle. As a result, it can be said that, as a candidate for potential bioremedial strain for extra-terrestrial applications, *P. fungorum* is able to withstand the harsh environmental parameters of a Mars-like environment and our findings indicate that this species is a suitable candidate for further experimentation.

When examining the combined effects of freeze-thaw and UV, it is important to note the doubling times of the test candidates. *D. radiodurans*, *P. cepacia* (a close relative to *P. fungorum*) and *E. coli* all have reported doubling times of 2.6 h, 3 h and 25 min, respectively [76,77,78]. While the induction of a stationary phase reduces the effects of UV damage on cells, it may also be possible that rapid doubling times allow for a greater level of DNA repair overall in response to environmentally-adverse effects, suggesting an explanation as to why both *E. coli* strains showed increased levels of viability, while both resistant strains (*D. radiodurans* and *P. fungorum*) showed a reduced viability with respect to the independent insults.

While UV resistance upon freezing and thawing appears to be strain-dependent, this phenomenon is useful for microbes intended for bioremediation in extra-terrestrial environments. There are no reported studies examining the phenotypic response of the selected strains to UVC energy over the course of 20 freeze-thaw cycles, thus highlighting a level of importance in furthering our understanding of these microbes both terrestrially and extra-terrestrially.

## 5. Conclusions

In this study *E. coli* ATCC strain 25922, *E. coli* ATCC strain 9079, *P. fungorum* DSM strain 17061 and *D. radiodurans* ATCC strain 13939 were subjected to three different rounds of experimentation to simulate Mars-like conditions. These included individual successive freezing and thawing experiments, with temperatures ranging from −80 °C to +30 °C, independent UVC dose-response experimentation and a combination of both freeze-thaw followed by UVC exposure. When exposed to these parameters separately, the test samples responded as detailed above, however, when combined, both species-dependent synergistic and antagonistic effects were observed.

While the results observed are species-dependent, the overall experimental data suggest that freeze-thawing significantly increases the UV resistance of both *E. coli* strains, while reducing the UVC-resistance of *P. fungorum* and *D. radiodurans*. It was also observed that *P. fungorum* DSM 17061 is a highly UV-resistant microbe. Given the resistant nature of the test microbes to adverse Mars-like conditions, these species are potential candidate bioremedial microbes for future potential employment on Mars, based upon their Earthly bioremedial capabilities.

Within an extra-terrestrial setting, these data suggest that a wide variety of common bacteria can survive the tested immediate harsh conditions found on the equatorial Mars-like landscape. Using the data obtained from this research, these selected microbes could potentially be capable of being strategically introduced to the future Martian landscape.
